# Sperm-Associated Antigen 9 Promotes Influenza A Virus-Induced Cell Death via the c-Jun N-Terminal Kinase Signaling Pathway

**DOI:** 10.1128/mbio.00615-22

**Published:** 2022-05-31

**Authors:** Rui Gui, Huabin Zheng, Liping Ma, Renyi Liu, Xian Lin, Xianliang Ke, Chang Ye, Xiaoqin Jian, Quanjiao Chen

**Affiliations:** a CAS Key Laboratory of Special Pathogens and Biosafety, Wuhan Institute of Virology, Center for Biosafety Mega-Science, CAS Center for Influenza Research and Early Warning, Chinese Academy of Sciences, Wuhan, China; b University of Chinese Academy of Sciences, Beijing, China; College of Veterinary Medicine, Cornell University

**Keywords:** PANoptosome, DNA-dependent activator of interferon-regulatory factor, Z-DNA binding protein 1, c-Jun N-terminal kinase, JNK, cell death, influenza A virus, sperm-associated antigen 9, SPAG9

## Abstract

Upon influenza A virus (IAV) infection, the IAV progeny ribonucleoprotein complex, with a defective viral genome, is sensed by DNA-dependent activator of interferon-regulatory factor (DAI). DAI initiates the recruitment of an array of proteins to form a multiprotein platform (PANoptosome), which triggers apoptosis, necroptosis, and pyroptosis during IAV infection. However, the mechanisms mediating the assembly of the PANoptosome are unclear. Here, we identified a scaffold protein, sperm-associated antigen 9 (SPAG9), which could interact with DAI to promote cell death during IAV infection. We further demonstrated that the cell death enhanced by SPAG9 was achieved through the DAI/SPAG9/c-Jun N-terminal kinase (JNK) axis, which could promote IAV-induced DAI-mediated cell death, including apoptosis, necroptosis, and pyroptosis. Our data further showed that the DAI/SPAG9/JNK signaling pathway enhanced the interactions among receptor-interacting serine/threonine kinase 1 (RIPK1), RIPK3, and DAI, thereby promoting IAV-induced PANoptosome formation. Overall, our study for the first time revealed a feed-forward circuit signaling pathway that enhanced IAV-induced DAI-mediated cell death, provided insights into the molecular mechanisms of cell death, and established therapeutic targets to address infectious and inflammatory diseases.

## INTRODUCTION

Influenza viruses, including influenza A virus (IAV), are globally circulating respiratory pathogens that cause 3 to 5 million severe cases of influenza infection and result in 290,000 to 650,000 deaths annually (1, 2). Therefore, influenza viruses are a serious global health threat and impose a tremendous economic burden worldwide (3, 4). Among the different types of influenza viruses, IAV is the most common and virulent (5, 6). Therefore, there is an urgent need to elucidate the detailed virulence features and pathogenesis of IAV infection to identify novel therapeutic opportunities.

Cell death and inflammatory responses are observed during the replication of IAV *in vitro* and *in vivo* (7–9). Excessive cell death and immune responses can also distinguish between mild and severe infections (10, 11). Moreover, excessive cell death within the upper and lower respiratory tracts and lung parenchyma further exacerbates inflammation, compromises the integrity of the epithelial cell barrier, and contributes to respiratory failure and even host death (12). Assembly of the PANoptosome (a multiprotein platform which triggers apoptosis, necroptosis, and pyroptosis) is crucial for IAV-triggered cell death and inflammatory reactions; however, its molecular basis is not entirely clear. Therefore, elucidation of the mechanisms mediating PANoptosome assembly is essential for improving our understanding of the pathogenesis of IAV infection (13, 14).

DNA-dependent activator of interferon (IFN) regulatory factor (DAI [ZBP1]) is a cytosolic DNA sensor that plays a vital role in inflammation activation, cell death, and host defense. DAI senses viral ribonucleoproteins (vRNPs) of IAV to recruit a series of key proteins, such as receptor-interacting serine/threonine kinase 1 (RIPK1), RIPK3, NLRP3, caspase-6, and caspase-8, to form large signaling complexes (e.g., the PANoptosome) and subsequently induces apoptosis, necroptosis, and pyroptosis (PANoptosis) (13, 15, 16). Since DAI plays a key role in cell death, we screened the cellular proteins that interact with DAI to identify novel proteins that contribute to PANoptosome formation (13). We found that the SPAG9 (JIP4) protein can interact with DAI and promote IAV-induced cell death. Moreover, we evaluated the molecular mechanisms mediating IAV-dependent cell death. Our findings provide important insights into potential targets that may be utilized for the development of anti-IAV therapies.

## RESULTS

### Screening of cellular proteins that interact with DAI.

**FIG 1:**
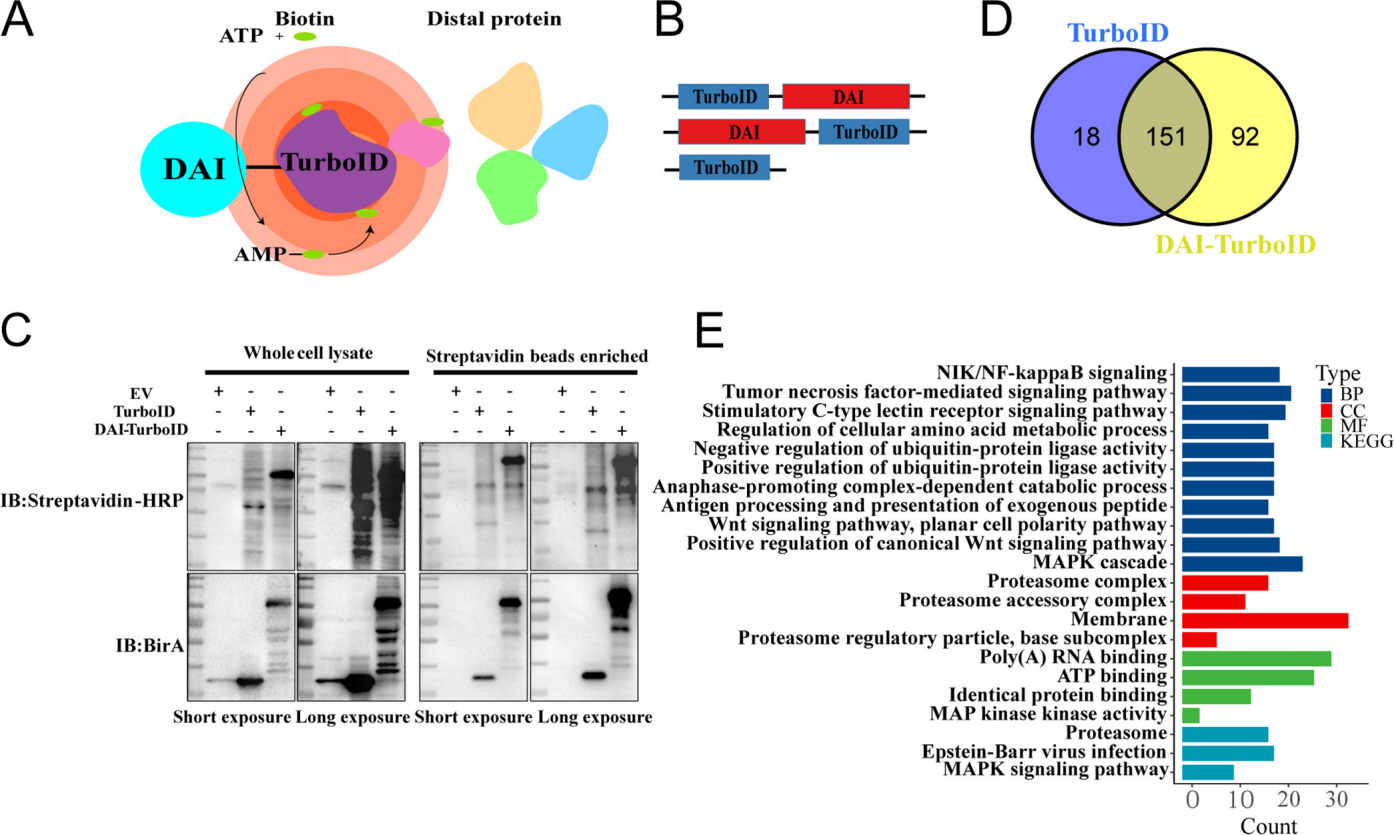
Identification of proximal and interacting proteins of DAI. (A) Schematic of the experimental design. TurboID catalyzes the formation of biotin-5′-AMP anhydride, which diffuses to biotinylate proximal endogenous proteins on lysine residues. (B) Diagram of the constructs used for the identification of proximal and interacting proteins of DAI. A flexible (GGGS)3 linker was used to link DAI and TurboID. (C) HEK293T cells were transfected with the indicated constructs or empty vector (EV). After 30 h, TurboID labeling was performed for 30 min with 500 μM biotin. Whole-cell lysates and streptavidin-enriched lysates were analyzed by streptavidin or anti-BirA (E. coli biotin ligase) blotting after live biotinylation. (D) Venn diagram depicting the proteins that interacted with TurboID (left) and/or DAI-TurboID (right). (E) GO (CC, cellular component; BP, biological process; MF, molecular function) and KEGG enrichment analysis of DAI TurboID hits (a total of 92 proteins). The count represents the number of the proteins enriched in the indicated signal pathway.

To identify new proteins involved in the PANoptosome, we used the TurboID technique, a novel proteomic technique that depends on nonspecific covalent proximity biotinylation of lysine residues derived from the Escherichia coli biotin ligase (BirA) (17). TurboID was fused to the C and N termini of DAI. Schematics depicting TurboID proximity labeling and DAI constructs are shown in Fig. 1A and B. To confirm whether the biotinylation specificity behaves differently when TurboID is fused to the N- or C-terminal part of the DAI, we first compared the biotinylation activities of TurboID-DAI and DAI-TurboID; no differences were observed (Fig. S1A).

10.1128/mbio.00615-22.1 FIG S1 Analysis and comparison of biotinylation and biological functions of DAI-TurboID and TurboID-DAI. (A) The indicated constructs or empty vector (EV) were transfected into WT HEK293T cells, and biotin (final concentration, 500 μM) was added at the indicated times for 30 min. Biotinylated proteins were detected by blotting with streptavidin-horseradish peroxidase-conjugated (streptavidin-HRP) antibodies. (B) WT HEK293T cells were transfected with the indicated constructs or EV together with an NF-κB reporter plasmid, and NF-κB-dependent luciferase activity was analyzed 24 h later. (C) WT HEK293T cells were transfected with the indicated constructs for 36 h, and anti-RIPK3 immunoprecipitation (IP) was performed to detect RIPK1, RIPK3, DAI-TurboID, or TurboID-DAI. (D) WT RAW264.7 cells were transfected with siRNA against indicated gene or nonspecific control (NC), and the mRNA levels of the indicated genes were detected by real-time RT-qPCR after 36 h. All experimental groups and the nonspecific control were compared to each other. (E) WT RAW264.7 cells were pretransfected with siRNAs against the indicated genes or nonspecific control and infected with IAV (PR8) at an MOI of 2. Cell death was assessed at 24 h postinfection. All experimental groups and the nonspecific control were compared to each other. *P* values were determined by Student's *t* test. *, *P* < 0.05; **, *P* < 0.01; ***, *P* < 0.001. Download FIG S1, TIF file, 0.9 MB. Copyright © 2022 Gui et al. 2022 Gui et al. https://creativecommons.org/licenses/by/4.0/ This content is distributed under the terms of the Creative Commons Attribution 4.0 International license.

We next compared the functionality of DAI in both the N- and C-terminal fusions with the TurboID domain by assessing its potential to activate NF-κB and to recruit RIPK1 and RIPK3. The NF-κB luciferase reporter activity of DAI-TurboID was slightly higher than that of TurboID-DAI (Fig. S1B).

RIPK1 and RIPK3 were recruited to DAI form the core components of the PANoptosome after DAI activation (18, 19). Therefore, we next compared the ability of TurboID-DAI and DAI-TurboID to interact with RIPK1 and RIPK3. As shown in Fig. S1C, the interaction of TurboID-DAI with RIPK1 and RIPK3 was comparable to that of DAI-TurboID. Thus, DAI-TurboID was chosen as an ideal candidate for the discovery of new proteins.

Biotinylated proteins were then detected by streptavidin blotting, and TurboID and DAI-TurboID expression levels were analyzed using anti-BirA antibodies at 30 h after transfection (Fig. 1C). Cell lysates, which contained biotinylated proteins, were enriched with streptavidin beads, and biotinylated proteins were highly enriched (Fig. 1C), extracted, and subjected to liquid chromatography-tandem mass spectrometry (LC-MS/MS) analysis. We identified 169 and 243 proteins in the TurboID and DAI-TurboID groups, respectively (Fig. 1D). Moreover, 92 proteins were shown to interact specifically with DAI-TurboID (Fig. 1D). Genes encoding these 92 proteins were then analyzed using Gene Ontology (GO) terms and the Kyoto Encyclopedia of Genes and Genomes (KEGG) database. The results showed that these genes were mainly related to NIK/NF-κB signaling, tumor necrosis factor (TNF) signaling, and the mitogen-activated protein kinase (MAPK) cascade (Fig. 1E). Based on bioinformatics analyses, we selected several proteins and examined their effects on IAV-induced cell death. Small interfering RNAs (siRNAs) whose silencing efficiency reached more than 70% were chosen to explore whether the corresponding protein affected IAV-mediated cell death (Fig. S1D). The siRNA sequences for the genes and nonspecific control siRNA are described in Table S1. The results indicated that knockdown of HECT, UBA, and WWE domain-containing E3 ubiquitin protein ligase 1 (HUWE1), mitogen-activated protein kinase kinase kinase kinase 4 (MAP4K4), sperm-associated antigen 9 (SPAG9), cullin-associated NEDD8-dissociated protein 1 (CAND1), and ubiquitin-specific peptidase 10 (USP10) affected IAV-mediated cell death (Fig. S1E). Among these targets, SPAG9 dramatically affected IAV-induced cell death, suggesting that SPAG9 may have crucial roles in IAV-triggered cell death.

10.1128/mbio.00615-22.5 TABLE S1 siRNAs against indicated genes or nonspecific control. Download Table S1, DOCX file, 0.01 MB. Copyright © 2022 Gui et al. 2022 Gui et al. https://creativecommons.org/licenses/by/4.0/ This content is distributed under the terms of the Creative Commons Attribution 4.0 International license.

### SPAG9 promoted IAV-triggered DAI-mediated cell death.

**FIG 2:**
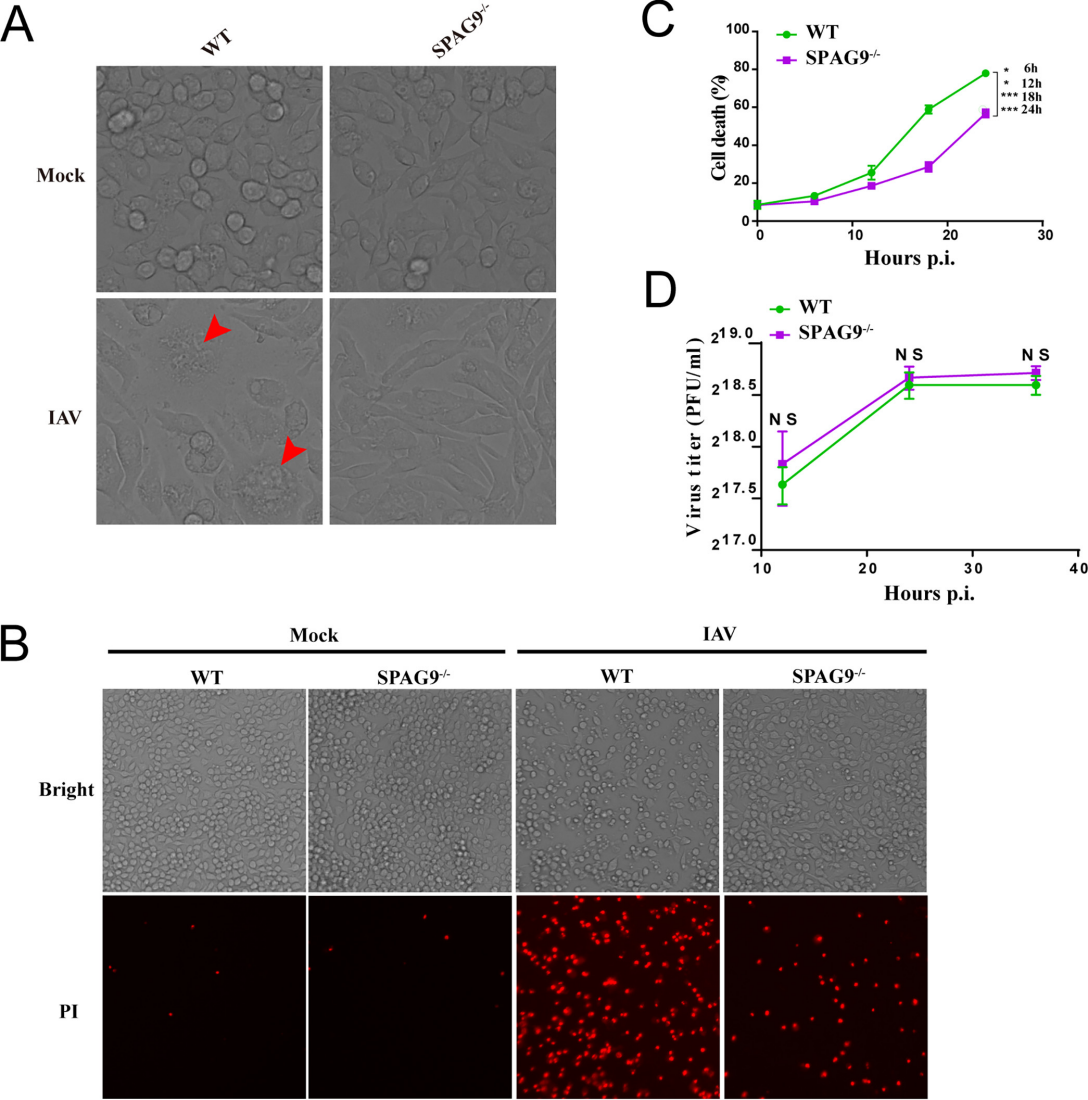
SPAG9 promoted IAV-induced cell death. (A) Cell death observed in bright-field microscopy (magnification, ×20). Arrowheads indicate dead cells. RAW264.7 cells were infected with IAV (PR8) at an MOI of 2 for 16 h. (B) Cell death was assessed by PI staining (magnification, ×10). RAW264.7 cells were infected with IAV (PR8) at an MOI of 2 for 12 h, and the cells were then stained with PI. (C) Quantification of cell death. WT or SPAG9^−/−^ RAW264.7 cells were infected with IAV (PR8) at an MOI of 2, and cell death was assessed at indicated times postinfection. (D) Effects of SPAG9 knockout on IAV replication. WT or SPAG9^−/−^ RAW264.7 cells were infected with IAV (PR8) at an MOI of 2, and supernatants were collected at the indicated time points postinfection for evaluation of virus titers using plaque assays. *P* values were determined by Student's *t* test. NS, not significant; *, *P* < 0.05; ***, *P* < 0.001.

As the core component of the PANoptosome, DAI is involved in initiation of PANoptosis following IAV infection. Thus, we investigated how SPAG9 affected DAI-mediated cell death during IAV infection. We utilized CRISPR gene editing to knock out SPAG9 in RAW264.7 macrophages (Fig. S2A) and found that cell death was reduced in SPAG9^−/−^ RAW264.7 macrophages during IAV infection (Fig. 2A and B). To confirm these finding, lactate dehydrogenase (LDH) release assays were used to quantitatively analyze cell death triggered by IAV infection. Our results showed that cell death was significantly reduced in SPAG9^−/−^ RAW264.7 macrophages compared with that in wild-type (WT) RAW264.7 macrophages (Fig. 2C), supporting a role of SPAG9 in IAV-mediated cell death.

10.1128/mbio.00615-22.2 FIG S2 SPAG9 was dispensable for apoptosis induced by UV radiation or etoposide and for necroptosis induced by TNF-α plus zVAD or LPS plus zVAD. (A) Immunoblot analysis of SPAG9 in RAW264.7 cells following CRISPR/Cas9 knockout. (B) RAW264.7 cells were exposed to UV radiation for 30 min, and cells were cultured for 10 h. Pro- and cleaved forms of caspase-3 were analyzed by immunoblotting (IB). (C) RAW264.7 cells were treated by etoposide (20 μM) for 12 h. Pro- and cleaved forms of caspase-3 were analyzed by IB. (D) RAW264.7 cells were exposed to UV radiation for 30 min, and cells were cultured for 10 h. Pro- and cleaved forms of caspase-8 were analyzed by IB. (E) RAW264.7 cells were treated by etoposide (20 μM) for 12 h. Pro- and cleaved forms of caspase-8 were analyzed by IB. (F and G) Phosphorylated MLKL was analyzed by IB in RAW264.7 cells treated with (F) TNF-α (10 ng/mL) plus zVAD (25 μM) or (G) LPS (150 ng/mL) plus zVAD (25 μM) for 10 h. Download FIG S2, TIF file, 2.1 MB. Copyright © 2022 Gui et al. 2022 Gui et al. https://creativecommons.org/licenses/by/4.0/ This content is distributed under the terms of the Creative Commons Attribution 4.0 International license.

SPAG9 has been reported to affect the replication of IAV (20). Therefore, to exclude the possibility that different cell death rates may be caused by distinct viral replication levels, a high multiplicity of infection (MOI) of 2 was used to maximize cell death independent of differences in viral replication. We performed a kinetic analysis of the viral replication in WT and SPAG9^−/−^ RAW264.7 macrophages following IAV infection in indicated time points (MOI = 2) and found that there were no differences in viral titers between WT and SPAG9^−/−^ RAW264.7 macrophages (Fig. 2D).

**FIG 3:**
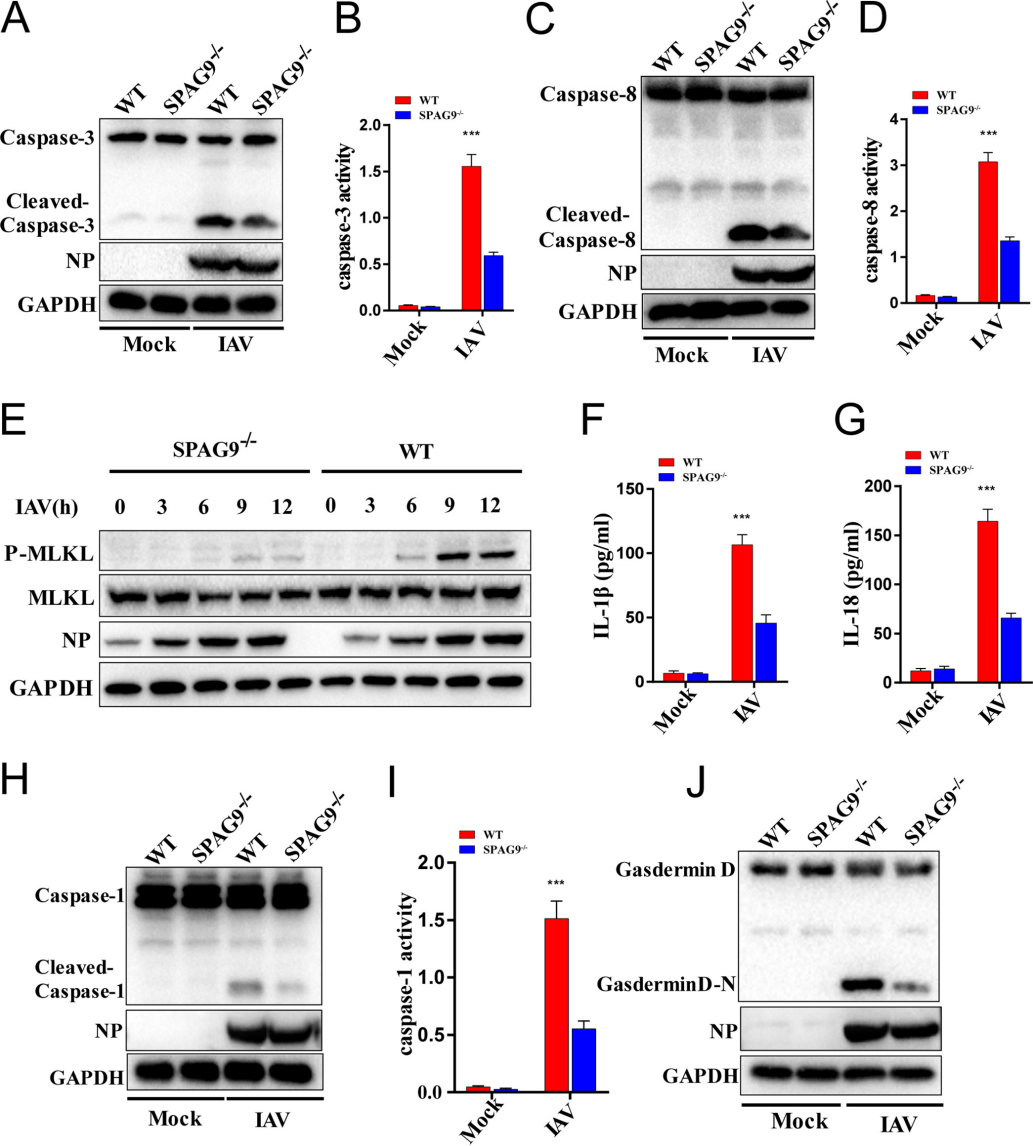
SPAG9 promoted IAV-induced apoptosis, pyroptosis, and necroptosis. (A) Apoptosis activation was evaluated by measuring cleaved-caspase-3 levels. WT and SPAG9^−/−^ RAW264.7 cells were infected with IAV (PR8) at an MOI of 2. Cells were collected at 16 h postinfection for immunoblot analysis. (B) Quantification of caspase-3 activity. RAW264.7 cells were infected with IAV (PR8) at an MOI of 2. Cells were collected at 16 h postinfection for ELISA. (C) Apoptosis activation was evaluated by measuring cleaved caspase-8 levels. WT and SPAG9^−/−^ RAW264.7 cells were assessed as described for panel A. (D) Quantification of caspase-8 activity. WT and SPAG9^−/−^ RAW264.7 cells were assessed as described for panel B. (E) Necroptosis activation was evaluated by measuring MLKL phosphorylation. (F) Quantification of IL-1β release by ELISA following IAV (PR8) infection. (G) Quantification of IL-18 release by ELISA following IAV (PR8) infection. (H) Pyroptosis activation was assessed by measuring cleaved caspase-1 levels. Pro- and cleaved forms of caspase-1 were analyzed by immunoblotting (IB) in RAW264.7 cells after infection with IAV (PR8) for 16 h. (I) Quantification of caspase-1 activity. (J) Pyroptosis activation was assessed by measurement of cleaved gasdermin D using immunoblotting (IB) in RAW264.7 cells after infection with IAV (PR8) for 16 h. *P* values were determined by Student's *t* test. ***, *P* < 0.001.

We then investigated the roles of SPAG9 in apoptosis, necroptosis, and pyroptosis upon viral infection. Apoptosis was assessed using caspase-3 and caspase-8. We observed that the activated initiator caspase-3 was downregulated (Fig. 3A and B), as was cleavage of executioner caspase-8, in SPAG9^−/−^ RAW264.7 macrophages after IAV infection (Fig. 3C and D). To further elucidate whether SPAG9 was specifically implicated in IAV-induced DAI-mediated apoptosis or globally regulated apoptosis, we treated WT and SPAG9^−/−^ RAW264.7 macrophages with UV radiation or etoposide. Apoptosis levels in WT and SPAG9^−/−^ RAW264.7 macrophages were similar (Fig. S2B-E). Taken together, these results showed that SPAG9 specifically regulated IAV-induced DAI-mediated apoptosis.

We then investigated the effects of SPAG9 on necroptosis. Unlike apoptosis and pyroptosis, which are induced by initiator caspases, necroptosis is triggered by RIPK3, which phosphorylates the pseudokinase mixed-lineage kinase domain-like (MLKL), leading to translocation of MLKL to the plasma membrane and subsequent induction of necroptosis (21–23). We found that MLKL phosphorylation was decreased in SPAG9^−/−^ RAW264.7 macrophages compared with that in wild-type (WT) RAW264.7 macrophages upon IAV infection (Fig. 3E). As a control, there were no differences in phosphorylated MLKL in WT and SPAG9^−/−^ RAW264.7 macrophages treated with the classical necroptotic inducer TNF-α plus Z-VAD-FMK (zVAD) or lipopolysaccharide (LPS) plus zVAD (Fig. S2F and G). These observations implied that SPAG9 specifically promoted IAV-induced DAI-mediated necroptosis.

We next evaluated pyroptosis in these cells. The results showed that interleukin 1β (IL-1β) and IL-18 release and activation of caspase-1 were decreased in SPAG9^−/−^ RAW264.7 macrophages, suggesting that pyroptosis was suppressed following IAV infection (Fig. 3F to I). These findings were further supported by the observation that gasdermin D (GSDMD) cleavage was also decreased in SPAG9^−/−^ RAW264.7 macrophages upon IAV infection (Fig. 3J). Overall, these results implied that SPAG9 played critical roles in IAV-induced cell death pathways, including apoptosis, necroptosis, and pyroptosis (PANoptosis).

### Inhibition of JNK signaling reduced IAV-induced cell death.

**FIG 4:**
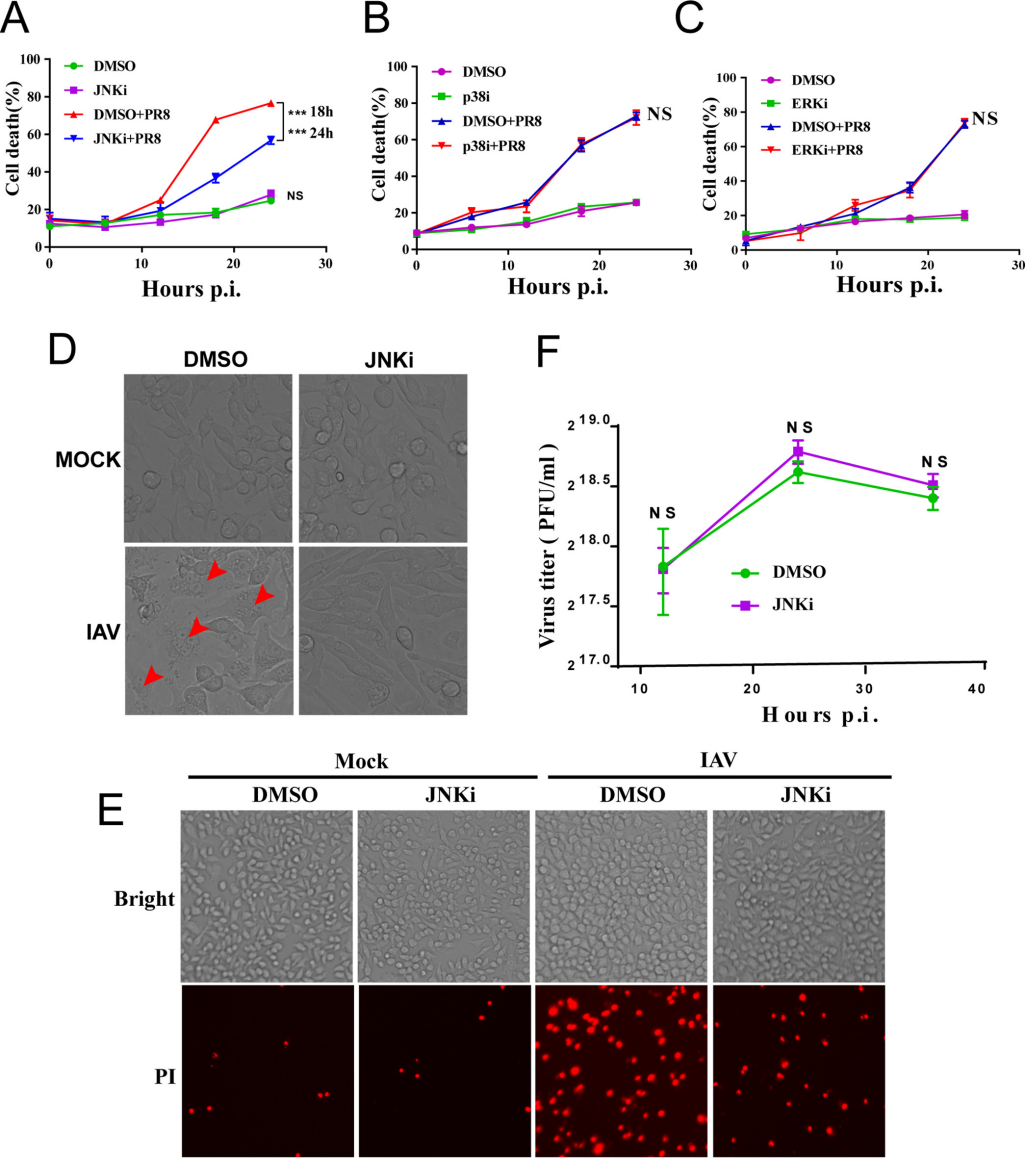
Inhibition of JNK signaling alleviated IAV-induced cell death. (A to C) Quantification of cell death. RAW264.7 cells were pretreated with JNK inhibitor (50 μM) (A), p38 inhibitor (20 μM) (B), and ERK inhibitor (20 μM) (C). RAW264.7 cells were infected with IAV (PR8) at an MOI of 2 for 1 h. Then, the maintenance medium with the indicated inhibitor was added. Cell death was assessed at the indicated times. (D) Cell death observed using bright-field microscopy (magnification, ×20). Cells were pretreated with 50 μM SP600125 for 1 h. RAW264.7 cells were infected with IAV (PR8) at an MOI of 2 for 16 h in culture medium containing the JNK inhibitor SP600125. Arrowheads indicate dead cells. (E) Cell death was assessed by PI staining (magnification, ×10). RAW264.7 cells were infected with IAV (PR8) at an MOI of 2 for 12 h, and the cells were then stained with PI. (F) Virus titers with and without JNK inhibitor treatment. WT RAW264.7 cells were infected with IAV (PR8) at an MOI of 2, and supernatants were collected at indicated time points postinfection for evaluation of virus titers using plaque assays. *P* values were determined by Student's *t* test. NS, not significant; ***, *P* < 0.001.

SPAG9, also known as c-Jun N-terminal kinase (JNK)-interacting protein 4 or JNK-associated leucine zipper protein, is a scaffold protein encoded by the *SPAG9* gene that mediates the interactions of mitogen-activated protein kinase kinases (MKKs) and can activate specific signaling pathways (24). Therefore, we speculated that SPAG9 may activate downstream MAPK signaling to promote IAV-induced cell death. To test this, we used three different inhibitors, i.e., SP600125 (a JNK signaling pathway inhibitor [JNKi]), SB203580 (a p38 signaling pathway inhibitor [p38i]), and U0126-EtOH (an extracellular signal-regulated kinase [ERK] signaling pathway inhibitor [ERKi]), to explore the involvement of MAPK pathways in IAV-induced cell death. As shown in Fig. 4A to C, only SP600125 suppressed IAV-mediated cell death. To further confirm that SP600125 blocked IAV-induced cell death, we performed microscopy and propidium iodide (PI) staining assays. The data showed that JNKi reduced IAV-mediated cell death (Fig. 4D and E).

**FIG 5:**
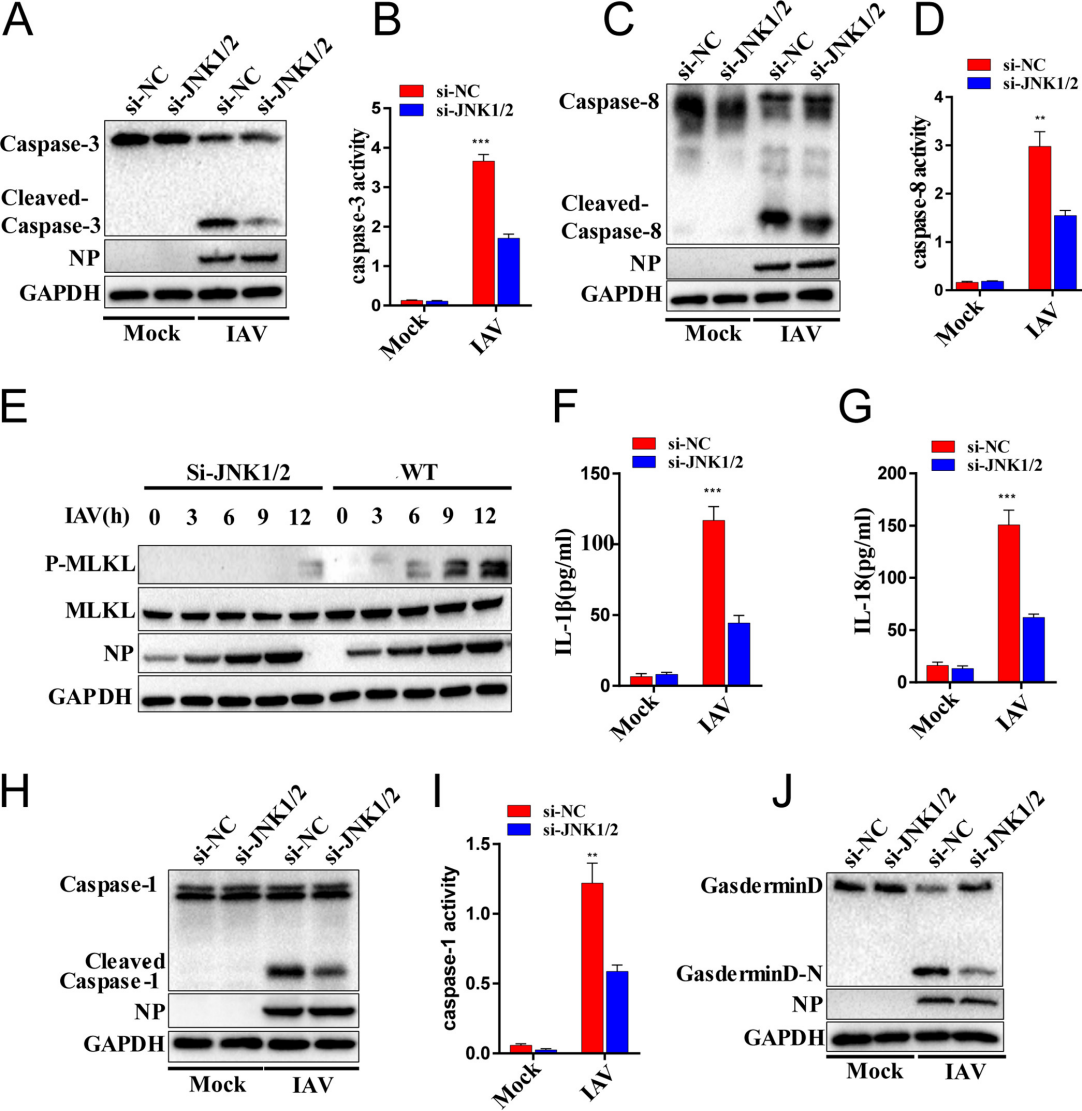
Inhibition of JNK signaling blocked IAV-induced apoptosis, pyroptosis, and necroptosis. RAW264.7 cells were infected with IAV (PR8) for 16 h, and cell lysates and supernatants were collected for testing. (A) Cleaved caspase-3 was evaluated by immunoblotting. (B) Caspase-3 activity was determined by ELISA. (C) Cleaved caspase-8 was analyzed by immunoblotting. (D) Caspase-8 activity was determined by ELISA. (E) Necroptosis activation was determined by measuring MLKL phosphorylation. (F) Quantification of IL-1β release by ELISA. (G) Quantification of IL-18 release by ELISA. (H) Caspase-1 was analyzed by immunoblotting. (I) Caspase-1 activity was assessed by ELISA. (J) Cleaved GSDMD was analyzed by immunoblotting. *P* values were determined by Student's *t* test. *, *P* < 0.05; **, *P* < 0.01; ***, *P* < 0.001.

A previous study showed that JNK could interfere with viral replication (25). To exclude the possibility that different cell death rates were caused by distinct amounts of viral particles, we used a high multiplicity of infection (MOI), i.e., 2, to maximize cell death independent of viral replication differences. We tested the viral replication in WT RAW264.7 macrophages (in the absence or presence JNKi) following IAV infection in indicated time points (MOI = 2). Notably, viral titers did not differ between the control and treated groups (Fig. 4F). We further elucidated the contribution of the JNK signaling pathway in each of these cell death pathways following IAV infection. We found that activated initiator caspase-3 (Fig. 5A and B) and activated executioner caspase-8 (Fig. 5C and D) were decreased by knockdown of JNK1/2 (Fig. S3A) after IAV infection, suggesting that apoptosis was suppressed.

10.1128/mbio.00615-22.3 FIG S3 The JNK pathway was dispensable for apoptosis induced by UV radiation or etoposide treatment and necroptosis induced by TNF-α plus zVAD or LPS plus zVAD. (A) WT RAW264.7 cells were transfected with siRNA against JNK1/2 or nonspecific control (NC), and JNK1/2 was analyzed by immunoblotting (IB) after 36 h. (B) RAW264.7 cells were exposed to UV radiation for 30 min, and cell were cultured for 10 h. Pro- and cleaved forms of caspase-3 were analyzed by IB. (C) RAW264.7 cells were treated with etoposide (20 μM) for 12 h. Pro- and cleaved forms of caspase-3 were analyzed by IB. (D) RAW264.7 cells were exposed to UV radiation for 30 min, and cells were cultured for 10 h. Pro- and cleaved forms of caspase-8 were analyzed by IB. (E) RAW264.7 cells were treated with etoposide (20 μM) for 12 h. Pro- and cleaved forms of caspase-8 were analyzed by IB. (F and G) Phosphorylated MLKL (p-MLKL) was analyzed by IB in RAW264.7 cells stimulated with (F) TNF-α (10 ng/mL) plus zVAD (25μM) or (G) LPS (150 ng/mL) plus zVAD (25 μM) for 10 h. Download FIG S3, TIF file, 1.9 MB. Copyright © 2022 Gui et al. 2022 Gui et al. https://creativecommons.org/licenses/by/4.0/ This content is distributed under the terms of the Creative Commons Attribution 4.0 International license.

To further evaluate whether the JNK signal was specifically implicated in IAV-induced DAI-mediated apoptosis or had a more global effect on apoptosis, we treated WT RAW264.7 macrophages with UV radiation or etoposide in the presence of JNKi. There were no differences in apoptosis rates between treated and control RAW264.7 macrophages following treatment with these classic apoptosis inducers (Fig. S3B to E). Taken together, these results showed that JNK signaling specifically modulated IAV-induced DAI-mediated apoptosis.

Next, we examined the roles of the JNK pathway in IAV-induced necroptosis. We found that MLKL phosphorylation was decreased in JNK1/2 knockdown cells after IAV infection (Fig. 5E). Furthermore, the necroptosis (induced by TNF-α plus zVAD or LPS plus zVAD) observed in SP600125-treated cells was indistinguishable from that in untreated control cells (Fig. S3F and G), indicating that JNK signaling promoted IAV-induced necroptosis.

To explore the contribution of the JNK pathway in IAV-induced pyroptosis, JNK1 and -2 were knocked down, and cells were infected with IAV. The results demonstrate that JNK1/2 knockdown decreased pyroptosis by reducing IL-1β and IL-18 release and activating caspase-1 (Fig. 5F to I). We also observed decreased GSDMD cleavage in IAV-infected cells (Fig. 5J). Taken together, these data implied that the JNK pathway was involved in multiple IAV-induced cell death pathways, including apoptosis, necroptosis, and pyroptosis.

### Activation of JNK signaling by the DAI/SPAG9 axis.

**FIG 6:**
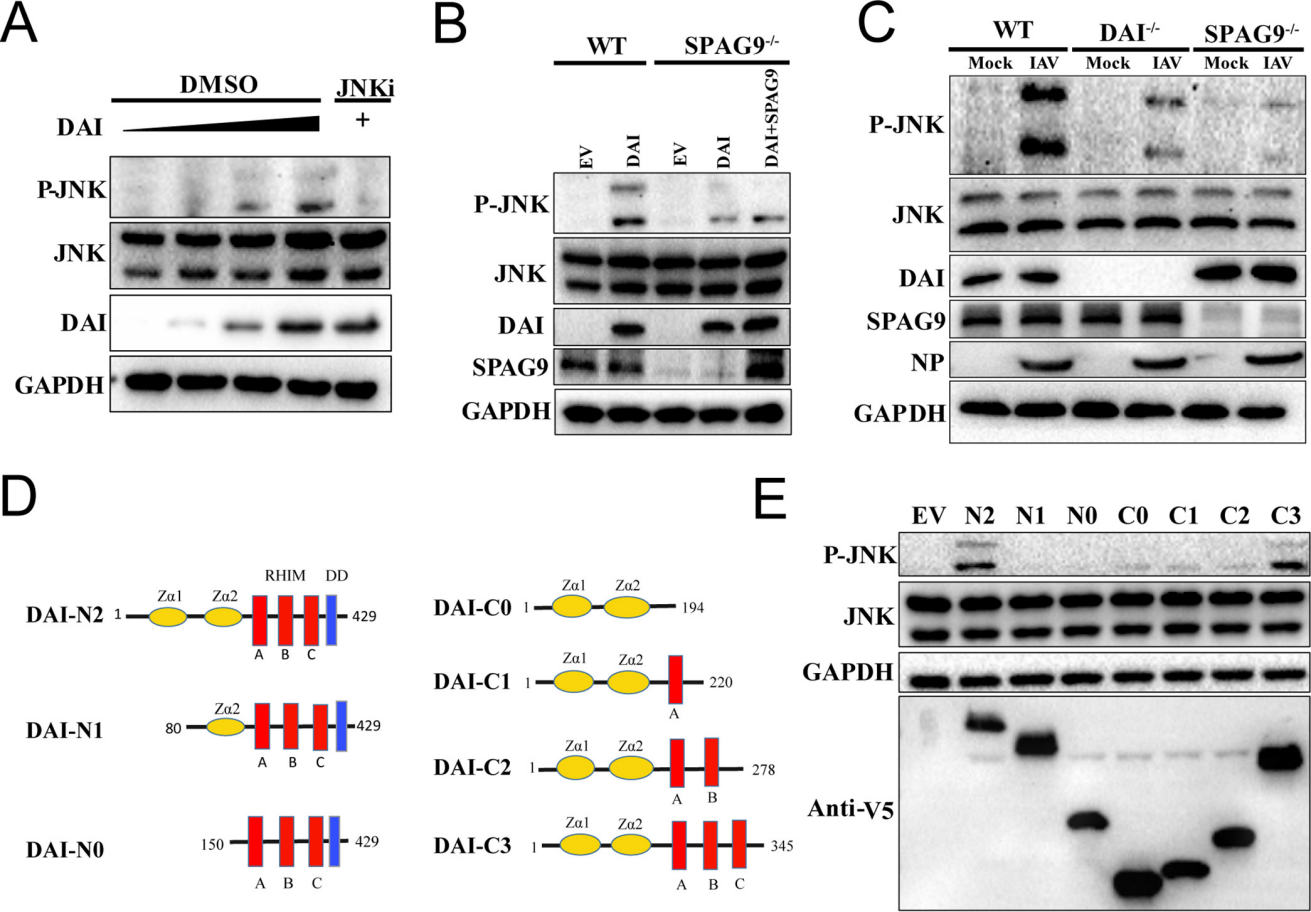
IAV infection activated JNK via the DAI/SPAG9/JNK pathway. (A) HEK293T cells were transfected with increasing concentrations of DAI or treated with the JNK inhibitor SP600125 (50 μM) as a negative control. Phosphorylated and total JNK1/2 were analyzed by immunoblotting. (B) Wild-type (WT) or SPAG9^−/−^ HEK293T cells were transfected with the indicated plasmids, and phosphorylated and total JNK1/2 were analyzed by immunoblotting. (C) WT, DAI^−/−^, and SPAG9^−/−^ RAW264.7 cells were infected with IAV (PR8; MOI = 2), and phosphorylated and total JNK1/2 were analyzed by immunoblotting. (D) Domain architecture of DAI and schematic of DAI constructs used in this study. (E) HEK293T cells were transfected with the indicated DAI constructs, and phosphorylated and total JNK1/2 were analyzed by immunoblotting.

RIG-I-like receptors and Toll-like receptors have been implicated in the activation of the JNK signaling pathway upon IAV infection (26). SPAG9 acts as a scaffolding protein, providing a platform for MKKs and their targets to activate the JNK signaling pathway (27). Our experiments demonstrated that SPAG9 may be involved in the PANoptosome by proximity labeling with DAI-TurboID. Therefore, we assumed that JNK signaling was activated via the DAI/SPAG9 cascade. As expected, as the expression of DAI increased in WT HEK293T cells, JNK was activated in a concentration-dependent manner (Fig. 6A).

To further confirm these findings, DAI or SPAG9 and DAI were overexpressed in SPAG9^−/−^ HEK293T cells. Importantly, JNK phosphorylation was reduced in overexpressed DAI SPAG9^−/−^ HEK293T cells compared with that in cells showing co-overexpression of SPAG9 and DAI (Fig. 6B). Furthermore, in SPAG9^−/−^ HEK293T cells that overexpress DAI and SPAG9, the phosphorylated JNK was partially restored (Fig. 6B). Moreover, evaluation of JNK phosphorylation in WT RAW264.7 cells, SPAG9^−/−^ RAW264.7 cells, and DAI^−/−^ RAW264.7 cells following IAV infection demonstrated that knockdown of SPAG9 or DAI attenuated JNK phosphorylation (Fig. 6C). Taken together, these results implied that the JNK signaling pathway was activated via the DAI/SPAG9 axis following IAV infection.

To explore which regions of DAI were important for activating the JNK signaling pathway, we constructed a series of mutant DAI vectors according to their functional domains. As shown in Fig. 6D, the N-terminal Zα domain and C-terminal RIPK homotypic interaction motif domain were indispensable and the C-terminal death domain was dispensable for DAI-dependent activation of JNK (Fig. 6E).

### SPAG9 bound to DAI to enhance the interactions among RIPK1, RIPK3, and DAI.

**FIG 7:**
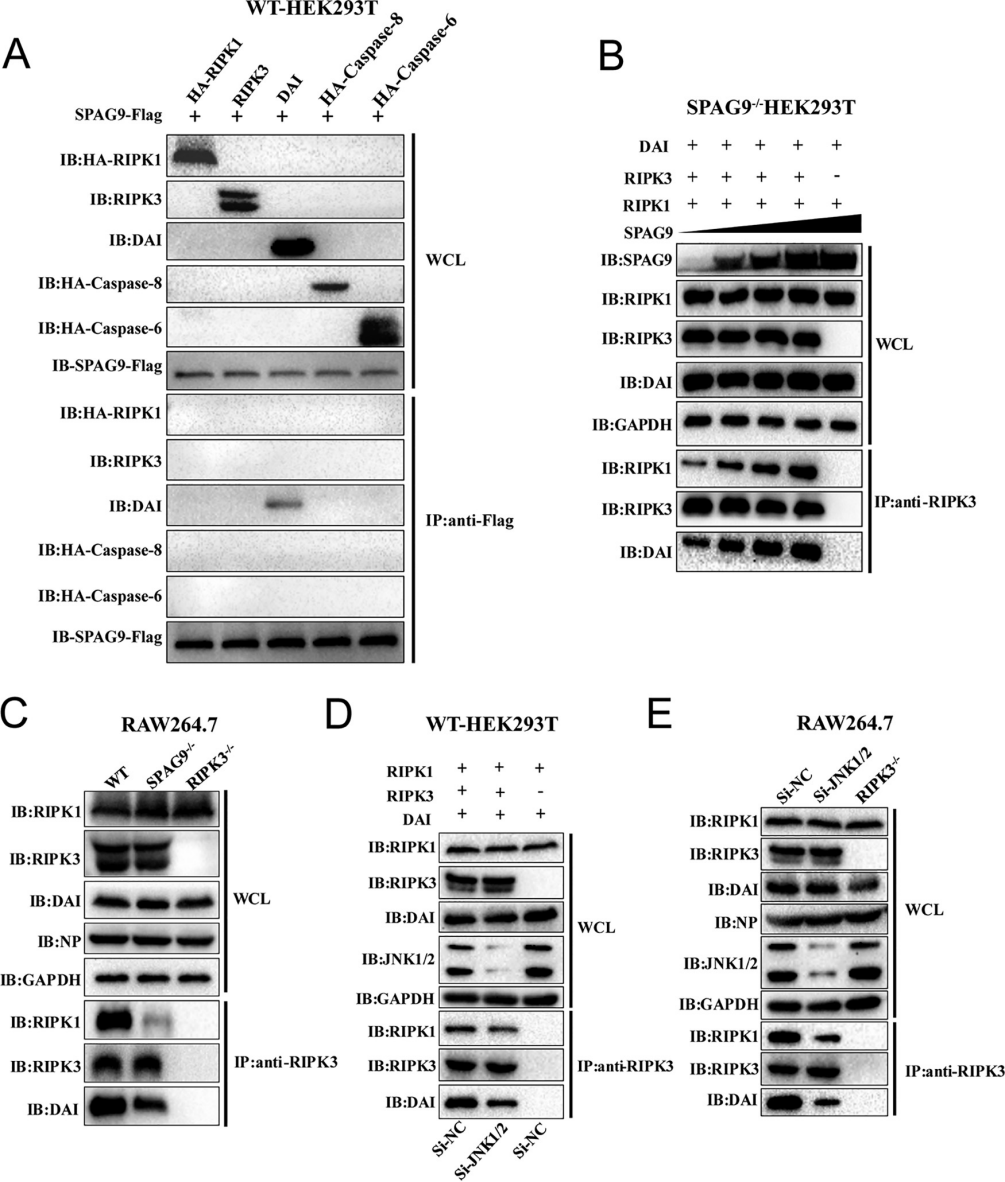
The SPAG9-JNK1/2 pathway is critical for enhancing interactions among RIPK1, RIPK3, and DAI. (A) Wild-type (WT) HEK293T cells were cotransfected with SPAG9-Flag and HA-RIPK1, RIPK3, DAI, HA–caspase-6, or HA–caspase-8 for 36 h. Anti-Flag immunoprecipitation was performed to detect RIPK1, RIPK3, DAI, HA–caspase-6, or HA–caspase-8. (B) SPAG9^−/−^ HEK293T cells were cotransfected with RIPK1, RIPK3, DAI, and increasing concentrations of SPAG9-Flag for 36 h, and anti-RIPK3 immunoprecipitation was performed to detect RIPK1, RIPK3, or DAI. (C) WT, SPAG9^−/−^, and RIPK3^−/−^ RAW264.7 cells were infected with IAV (PR8; MOI = 2) for 12 h, and anti-RIPK3 immunoprecipitation was performed to detect RIPK1, RIPK3, or DAI. (D) WT HEK293T cells were transfected with siRNA against JNK1/2 (25 nM), and at 24 h posttransfection, the cells were cotransfected with RIPK1, RIPK3, and DAI transfection siRNA for 36 h. Anti-RIPK3 immunoprecipitation was performed to detect RIPK1, RIPK3, and DAI. (E) WT and RIPK3^−/−^ RAW264.7 cells were transfected with siRNA against JNK1/2 (25 nM), and after 24 h posttransfection, the cells were infected with IAV (PR8; MOI = 2). After 12 h, anti-RIPK3 immunoprecipitation was performed to detect RIPK1, RIPK3, or DAI. IB, immunoblotting.

Because SPAG9 regulated all three cell death pathways (PANoptosis) through the JNK signaling pathway, we next hypothesized that the SPAG9/JNK axis may act on the PANoptosome to regulate IAV-induced cell death. To evaluate the recruitment of SPAG9 to the PANoptosome, we overexpressed SPAG9 plus RIPK1, RIPK3, DAI, caspase-8, and caspase-6, which are components of the PANoptosome, and assessed the interactions among these proteins. DAI specifically bound to SPAG9 (Fig. 7A), suggesting that SPAG9 may be recruited to the PANoptosome by DAI to activate JNK signaling and modulate the interactions of RIPK1, RIPK3, and the DAI complex. To verify this, RIPK1, RIPK3, and DAI were coexpressed in SPAG9^−/−^ HEK 293T cells with increasing concentrations of SPAG9. The amounts of RIPK1 and DAI coimmunoprecipitated by RIPK3 increased as the concentration of SPAG9 increased (Fig. 7B), implying that SPAG9 could promote the interactions among RIPK1, RIPK3, and DAI. Furthermore, coimmunoprecipitation under endogenous conditions in WT and SPAG9^−/−^ RAW264.7 macrophages demonstrated that the absence of SPAG9 reduced the amount of RIPK1 and DAI coimmunoprecipitated with RIPK3 (Fig. 7C). RIPK3^−/−^ RAW264.7 macrophages infected with IAV were used as a negative control. To determine whether JNK signaling was involved in the interactions among RIPK1, RIPK3, and DAI, these three proteins were coexpressed in JNK1/2 knockdown WT HEK293T cells. Our results showed that the amounts of RIPK1 and DAI coimmunoprecipitated with RIPK3 were obviously decreased compared with those in the control group, suggesting that JNK signaling enhanced the interactions among RIPK1, RIPK3, and DAI (Fig. 7D). These results were further verified by endogenous coimmunoprecipitation in RAW264.7 macrophages (Fig. 7E).

Next, we compared the interactions among RIPK1, RIPK3, and DAI in the presence of p38i and ERKi. As expected, no differences were found between the two groups (Fig. S4B and C), further confirming that JNK signaling mediated the interactions among RIPK1, RIPK3, and DAI.

10.1128/mbio.00615-22.4 FIG S4 ERKi and p38i treatment did not affect the interactions among RIPK1, RIPK3, and DAI. (A) RIPK3 in RAW264.7 cells was analyzed by immunoblotting (IB) following CRISPR-directed deletion. (B and C) RIPK3^−/−^ and WT RAW264.7 cells were infected with IAV (PR8; MOI = 2) following pretreatment with p38i (B) or ERKi (C) for 1 h. After 12 h, anti-RIPK3 immunoprecipitation (IP) was performed to detect RIPK1, RIPK3, and DAI. Download FIG S4, TIF file, 0.6 MB. Copyright © 2022 Gui et al. 2022 Gui et al. https://creativecommons.org/licenses/by/4.0/ This content is distributed under the terms of the Creative Commons Attribution 4.0 International license.

## DISCUSSION

Sensing of vRNP by DAI can trigger the recruitment of a series of downstream proteins to form a multiprotein complex, leading to cell death (13, 28). Currently, little is known about the assembly of multiprotein complexes. Caspase-6 was the first caspase discovered to be involved in regulating PANoptosome assembly and promoting cell death by enhancing RIPK1, RIPK3, and DAI interaction (14). However, the other proteins engaging in complex assembly mediation are yet to be discovered. In this study, we found that SPAG9 was involved in DAI-mediated cell death via activation of the JNK pathway following IAV infection. Moreover, the SPAG9/JNK axis was shown to be dispensable for apoptosis and necroptosis triggered by classical stimuli. Simultaneously, we showed that the SPAG9/JNK axis facilitated pyroptosis upon IAV infection. However, because of a lack of suitable stimuli to induce pyroptosis, it is still unclear whether the SPAG9/JNK axis was specifically engaged in DAI-mediated pyroptosis following IAV infection.

SPAG9 was first identified from a human testis cDNA library as a member of the CT (cancer testis) antigen family in 1998 and was later found to be involved in the regulation of a variety of tumor invasion and migration factors and viral replication via MAPK signaling (27, 29, 30). Our data indicated that only JNK signaling was involved in DAI-mediated cell death following IAV infection. JNK is a highly conserved serine/threonine protein kinase that was first identified in 1990 in studies using the protein synthesis inhibitor cycloheximide and has since been shown to have critical roles in signal transduction (31, 32). The JNK signaling pathway functions by activating c-Jun N-terminal kinase phosphorylation (33). Upon IAV infection, multiple signaling pathways (RIG-I/Toll-like receptors) are involved in the activation of the JNK signaling pathway (26). We found here that IAV infection induced DAI-mediated JNK activation. However, additional studies are needed to address the functions of the JNK pathway in promoting the interactions among components of the RIPK1/RIPK3/DAI complex. Our findings suggested that SPAG9 mechanistically interacted with DAI and activated JNK signaling, thereby enhancing the interactions among RIPK1, RIPK3, and DAI following IAV infection. In the absence of SPAG9 or treatment with JNKi, the interactions among RIPK1, RIPK3, and DAI were suppressed upon IAV infection, particularly interactions involving RIPK1. Therefore, we hypothesized that DAI recruited SPAG9 to activate JNK and enhance the interactions among these three proteins, forming a positive feedback loop.

The binding of RIPK3 with DAI is dramatically enhanced in RIPK1-deficient cells, and previous studies in RIPK1^E-KO^ mice or in humans with biallelic RIPK1 mutations have shown that cell death and inflammation are dependent on the DAI/RIPK3 complex (34, 35). RIPK1 may also counteract cell death and inflammation mediated by the DAI/RIPK3 complex. However, our results showed that inhibition of JNK signaling notably reduced the interactions between RIPK1 and DAI and affected DAI-mediated cell death. One possible explanation is that RIPK1 may have both positive and negative roles in DAI/RIPK3 complex-triggered cell death (36). For example, RIPK1 may counteract the interaction between RIPK3 and DAI to inhibit cell death. Alternatively, when DAI is activated by its ligands and recruits RIPK3 to form the DAI-RIPK3 complex, RIPK1 may facilitate DAI/RIPK3 assembly and promote DAI-mediated cell death. Further studies are needed to assess the complex relationships among RIPK1, RIPK3, and DAI.

Taken together, our findings reveal a new function of SPAG9, as a critical immune regulator, engaging in DAI-mediated JNK signal pathway activation and cell death. At the molecular level, SPAG9 was found to be involved in the formation of the PANoptosome via DAI, which activated JNK signaling and formed a positive feedback loop to enhance the interactions among RIPK1, RIPK3, and DAI. These results broadened our understanding of the mechanisms through which the DAI/SPAG9/JNK signaling pathway regulates PANoptosome assembly and may establish a rationale for the development of therapeutics to treat diseases related to cell death.

## MATERIALS AND METHODS

### Cell culture.

RAW264.7 (ATCC TIB-71), HEK293T (ATCC CRL-3216), and MDCK (ATCC CCL-34) cells were grown in Dulbecco’s modified Eagle medium (GIBCO) contained 10% fetal bovine serum (FBS) (GIBCO) and incubated at 37°C in 5% CO_2_.

### Viruses and plasmids.

IAV (A/Puerto Rico/8/34 [H1N1, PR8]) was obtained from the Virus Resource Center of the Wuhan Institute of Virology of the Chinese Academy of Sciences and propagated by allantoic inoculation of 9- to 11-day-old chicken embryos with diluted (1:10^6^) seed virus. Stock virus titers were determined by plaque assays in MDCK cells.

Expression plasmids for DAI-TurboID-Flag, RIPK3, Flag-RIPK3, SPAG94-3xFlag, DAI, and HA–caspase-6 were constructed by inserting the coding sequences into plasmid pCDNA3.1(−), whereas the expression plasmid for HA-RIPK1 was constructed by inserting the coding sequence into pCAGGS. Plasmids pCDNA3.1(−) and pCAGGS were kind gifts from Hanzhong Wang (Wuhan Institute of Virology, CAS). pRK-HA-caspase-8 was kindly provided by Ke Peng (Wuhan Institute of Virology, CAS). The target sequences for single guide RNA (sgRNA)-SPAG9 (humans), sgRNA-SPAG9 (mice), sgRNA-DAI (mice), and sgRNA-RIPK3 (mice) were as follows: 5-TGTGAGAAAGATGTGCTGCA-3′, 5-TTGAACCTTTATATCCACTG-3′, 5-AGTCCTTTACCGCCTGAAGA-3′, and 5-AGTTCTTCACGGCTCACCAG-3′. The sgRNA was cloned into lentiCRISPR-v2 (Addgene 52961). Lentiviruses were produced in HEK293T by transfection of lentiCRISPR v2 together with psPAX2 (Addgene 12260) and pMD.2G (Addgene 12259).

### Transfection, antibodies, and reagents.

Antibodies against SPAG9 (catalog no. ab12331) were purchased from Abcam. Anti-DAI (catalog no. sc-271483) and anti-NP (catalog no. sc-101352) antibodies were purchased from Santa Cruz Biotechnology. Anti-DAI (catalog no. 13285-1-AP) and anti-RIPK3 (catalog no. 17563-1-AP) antibodies were purchased from Proteintech. Anti-MLKL (catalog no. A19685), anti-RIPK1 (catalog no. A19580), anti-glyceraldehyde 3-phosphate dehydrogenase (catalog no. AC002), anti-caspase-1 (catalog no. A0954), anti-GSDMD (catalog no. A18281), anti-caspase-8 (catalog no. A0215), and antihemagglutinin (anti-HA) (catalog no. AE008) antibodies were purchased from Abclonal. Anti-phospho-MLKL (catalog no. 37333S), anti-JNK (catalog no. 9252), and anti-phospho-JNK (catalog no. 9255S) antibodies were obtained from Cell Signaling Technology. Anti-Flag (catalog no. F1804-200UG) antibodies were purchased from Sigma-Aldrich. Anti-BirA (catalog no. 11582-RP01-100) antibodies were purchased from Sinobiological.

HEK293T cells were transfected using Lipofectamine 8000 (catalog no. C0533FT) purchased from Beyotime. The pan-caspase inhibitor Z-VAD-FMK (catalog no. S7023), JNK1/2/3 inhibitor SP600125 (catalog no. S1460), MEK1/2 inhibitor U0126-EtOH (S1102), and p38 inhibitor SB203580 (catalog no. S1076) were purchased from Selleck. LPS (catalog no. 82857-67-8), TNF-α (catalog no. T7539), biotin (catalog no. 58-85-5), and propidium iodide (PI) (catalog no. 25535-16-4) were purchased from Sigma-Aldrich.

### Generation of cell lines by lentiviral infection.

The indicated sgRNA (in lentiCRISPR-v2) and two other packaging plasmids (psPAX2 and pMD.2G) were transfected into HEK293T cells at ~80% confluence using Lipofectamine 8000. Lentivirus-containing cell culture media were collected 60 h after transfection and filtered through a 0.45-μm filter. The harvested lentivirus was added to fresh cell culture at ~50% confluence. At 48 h after infection, cells were transferred to complete fetal bovine serum-supplemented culture medium containing puromycin for selection, and single clones were then isolated and expanded.

### Propidium iodide staining.

RAW264.7 cells (1 × 10^6^) were seeded into 12-well plates. Cells were pretreated with the indicated reagent for 1 h, infected with virus for the indicated time in the presence of the indicated reagent, and stained with PI in culture medium (50-μg/mL final concentration; 10 min). The stained cells were visualized using a fluorescence microscope with excitation at 488 nm and emission at 635 nm. Data are representative of results from three independent experiments.

### TurboID labeling assay and TurboID pulldown.

HEK293T cells were cultured in Dulbecco’s modified Eagle medium containing 10% fetal bovine serum in a humidified atmosphere containing 5% CO_2_ at 37°C. The TurboID fusion construct of interest was transfected into HEK293T cells using Lipofectamine 8000. Biotin (final concentration, 500 μM) was added to the cell culture medium and incubated for 30 min (5% CO_2_, humidified at 37°C) after transfection for 30 h. After being washed three times with phosphate-buffered saline, the cells were lysed using lysis buffer (50 mM Tris-HCl [pH 7.4], 150 mM NaCl, 1% NP-40, 0.1% sodium dodecyl sulfate). To enrich biotinylated proteins from cell lysates, 100 μL streptavidin-coated agarose beads (Thermo Fisher Scientific) was washed twice with radioimmunoprecipitation assay (RIPA) lysis buffer, and lysates containing ~2 mg protein were incubated with beads overnight at 4°C. Sequentially, the beads were washed three times with RIPA buffer and twice with 150 mM NaCl buffer. To determine successful enrichment of the biotinylated proteins, a tenth of the volume was removed for immunoblotting analysis, and the remaining beads were flash-frozen in liquid nitrogen and stored at −80°C for LC-MS/MS analysis.

### Immunoprecipitation and immunoblotting.

For immunoprecipitation, cell lysates were centrifuged, and the supernatants were incubated for 4 h at 4°C with 1.5 μg of the indicated primary antibodies on a rotor. Protein A/G Plus-agarose (Santa Cruz Biotechnology) was then added to the lysates and incubated for an additional 2 h on a rotor. Agarose beads were centrifuged and washed three times with lysis buffer. Immunoprecipitates were eluted in 2× Laemmli buffer, incubated for 10 min at 95°C, and then subjected to immunoblotting analysis.

### RT-qPCR.

For reverse transcription-quantitative real-time PCR (RT-qPCR), total RNA was extracted using TRIzol (Invitrogen, USA). Synthesis of cDNA was conducted using the HiScript III 1st Strand cDNA synthesis kit (Vazyme, Nanjing, China) as recommended by the product manufacturer. qPCR was conducted with 2× SYBR green kit (Vazyme, Nanjing, China). The indicated gene-specific primers are described in Table S2.

10.1128/mbio.00615-22.6 TABLE S2 Gene-specific primers. Download Table S2, DOCX file, 0.01 MB. Copyright © 2022 Gui et al. 2022 Gui et al. https://creativecommons.org/licenses/by/4.0/ This content is distributed under the terms of the Creative Commons Attribution 4.0 International license.

### ELISA.

IL-1β and IL-18 levels were measured using enzyme-linked immunosorbent assay (ELISA) kits (IL-1β kit from BioLegend, Nanjing, China; IL-18 kit from eBioscience, USA).

### LDH release assay.

At the indicated times after IAV infection, serum-free cell supernatants were collected. LDH release was assessed using an LDH assay kit (Beyotime) according to the manufacturer’s protocol.

### Plaque assay.

The titers of influenza A virus were determined by plaque assay on MDCK cells. MDCK cells were initially inoculated at a seeding density of 5 × 10^5^ cells per well in a 12-well plate. Then, 24 h later, confluent MDCK cells were infected for 1 h at 37°C with 500 μL of MEM containing a 10× gradient dilution of viral stock. After removal of the inoculum following 3 washes with phosphate-buffered saline (PBS), MDCK cells were overlaid with 1 mL MEM containing 1% agar, 0.3% bovine serum albumin (BSA), and 2 μg/mL TPCK (tosylsulfonyl phenylalanyl chloromethyl ketone)-treated trypsin, and cultured at 37°C for 2 days. Plaques were monitored and counted.

### Statistical analysis.

Statistical significance was determined using Student’s *t* test. Error bars represent standard deviations. Results with *P* values less than or equal to 0.05 were considered significant. Graphs were generated using GraphPad Prism software (version 7.0).

### Data availability.

All the recombinant plasmids in this study are available from the corresponding author on reasonable request.
