# Supplementary figures and images for: Sperm-Associated Antigen 9 Promotes Influenza A Virus-Induced Cell Death via the c-Jun N-Terminal Kinase Signaling Pathway
Source: mBio. 2022 May 31;13(3):e00615-22. doi: 10.1128/mbio.00615-22 (PMC9239253; doi:10.1128/mbio.00615-22)

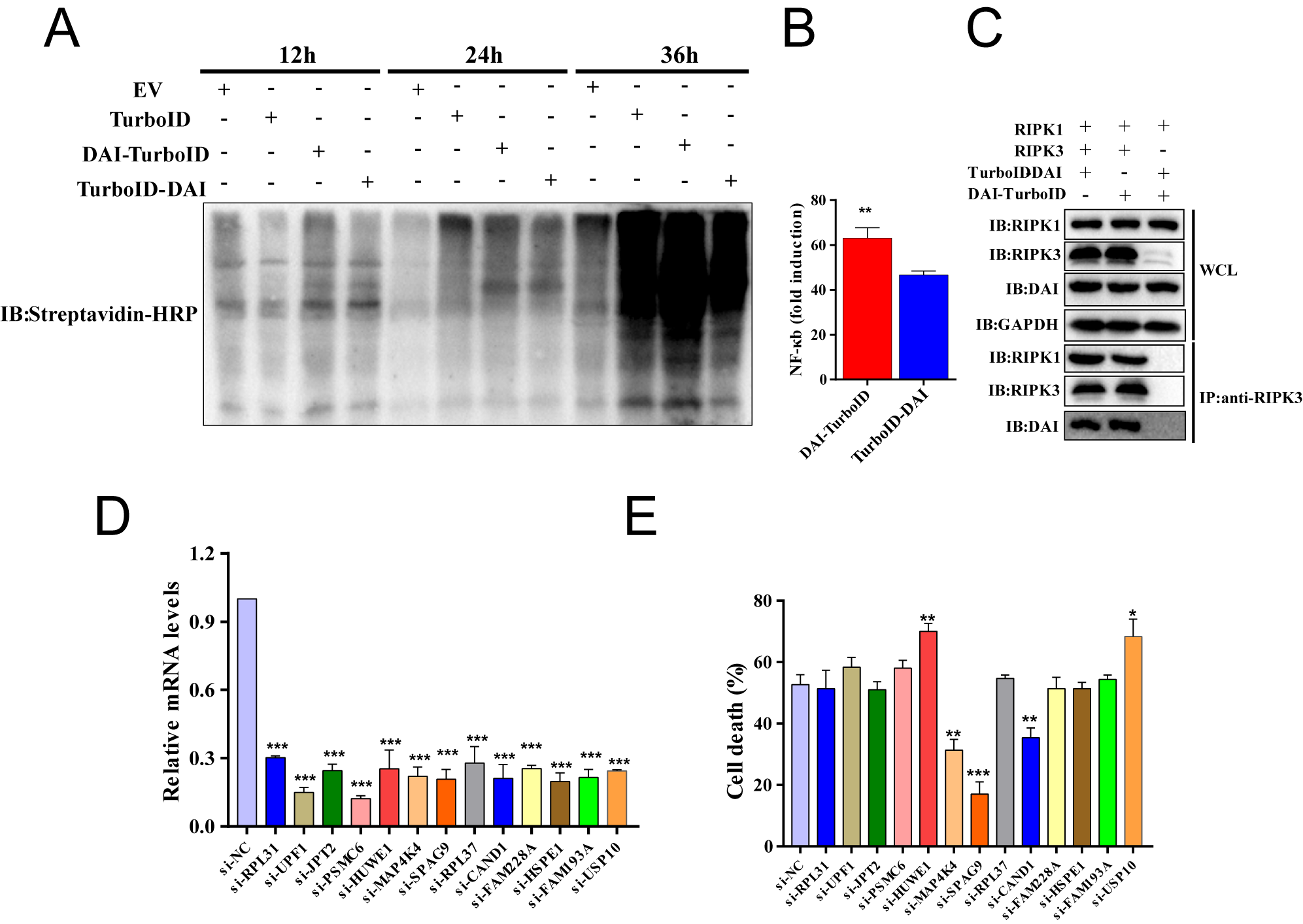

Supplement: FIG S1 [file mbio.00615-22-s0003.tif]

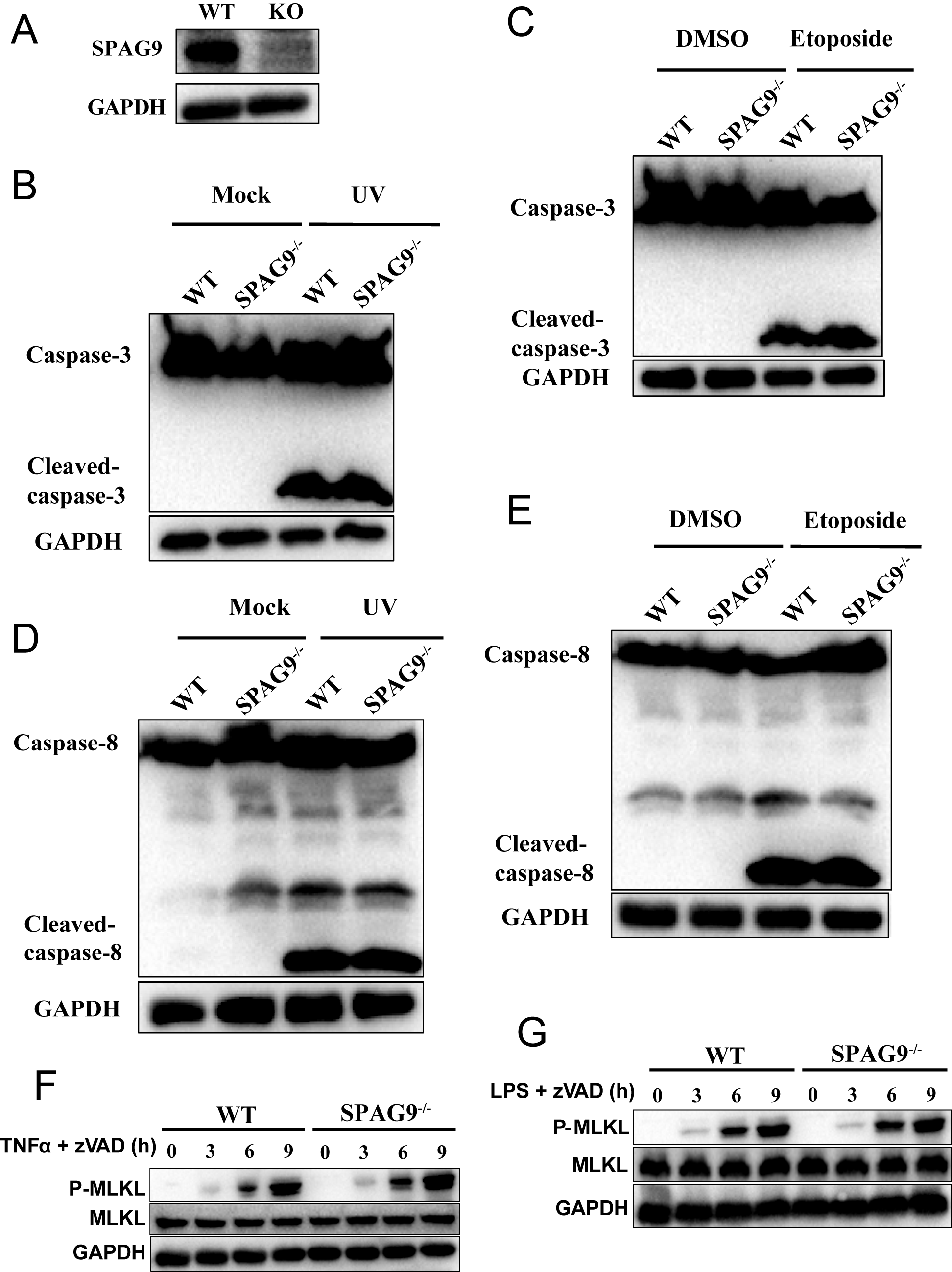

Supplement: FIG S2 [file mbio.00615-22-s0004.tif]

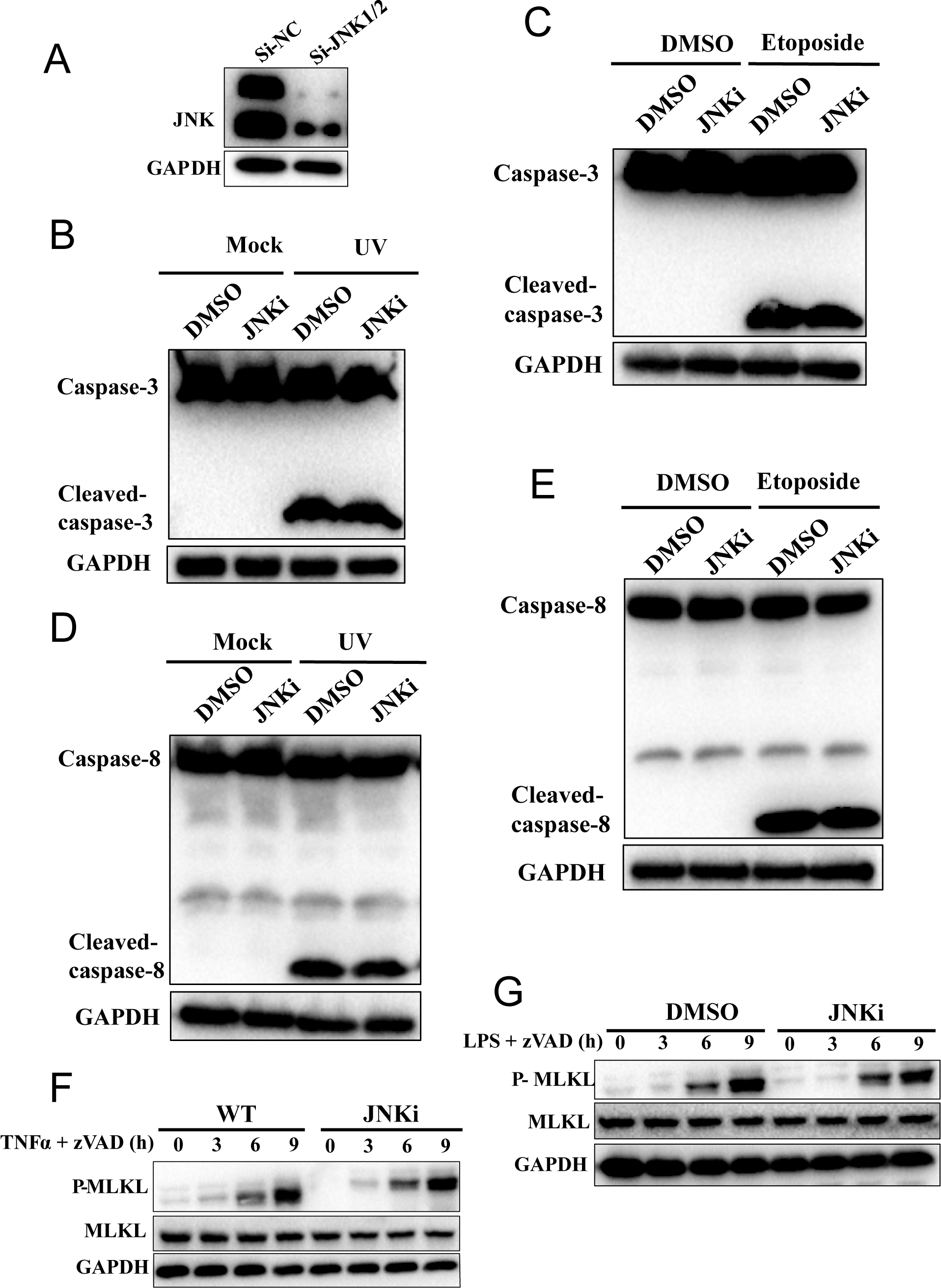

Supplement: FIG S3 [file mbio.00615-22-s0005.tif]

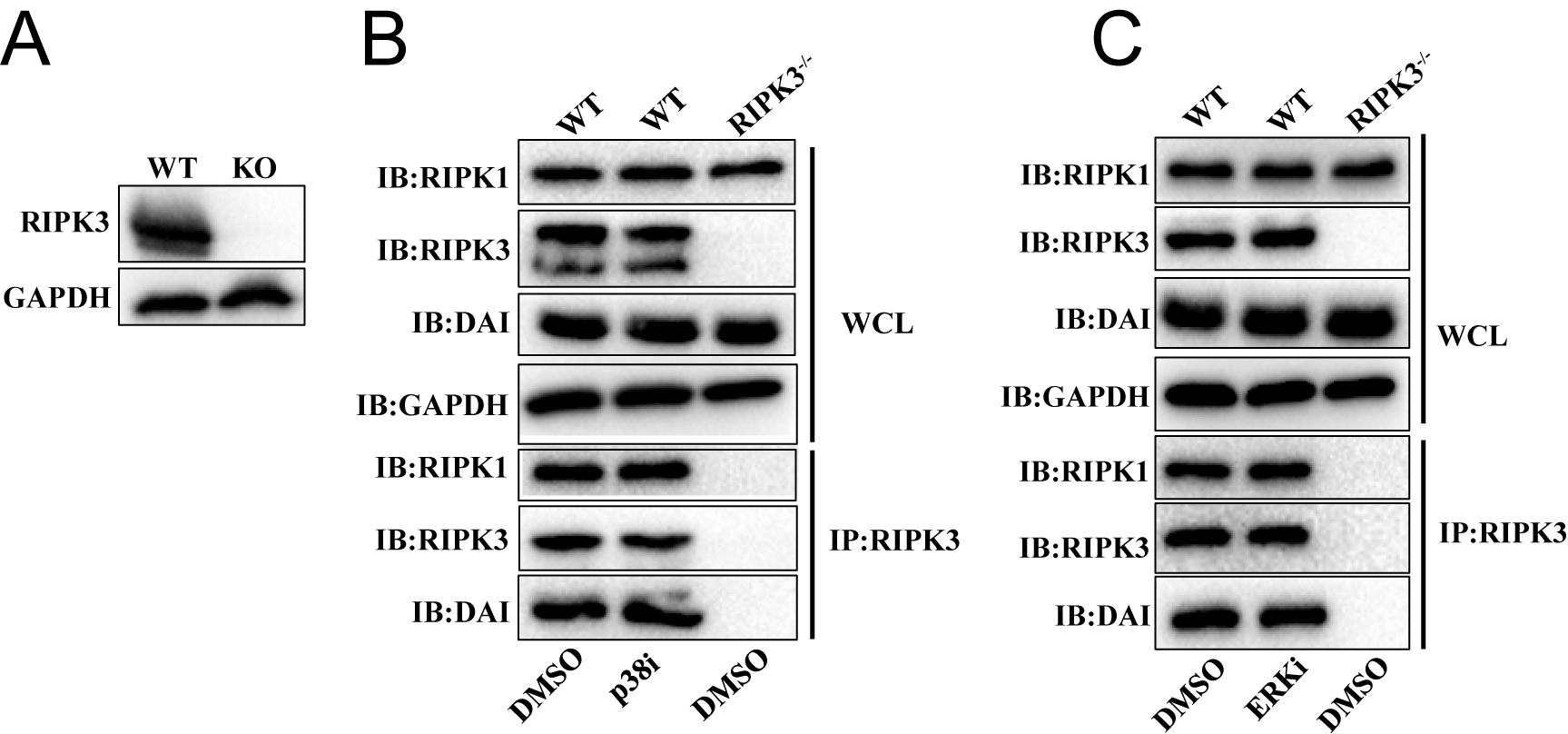

Supplement: FIG S4 [file mbio.00615-22-s0006.tif]
