# Supplementary material for: Sperm-Associated Antigen 9 Promotes Influenza A Virus-Induced Cell Death via the c-Jun N-Terminal Kinase Signaling Pathway
Source: mBio. 2022 May 31;13(3):e00615-22. doi: 10.1128/mbio.00615-22 (PMC9239253; doi:10.1128/mbio.00615-22)
Supplement: TABLE S1 [file mbio.00615-22-s0001.docx]

**Table S1.** The sequence of primers was used for RT-qPCR

| name | sequence |
| --- | --- |
| GAPDH-Forward (mouse) | 5'-TGGATTTGGACGCATTGGTC-3' |
| GAPDH-Reverse (mouse) | 5'-TTTGCACTGGTACGTGTTGAT-3' |
| UPF1-Forward (mouse) | 5'-GTGGCAGCCCCTAATCCAG-3' |
| UPF1-Reverse (mouse) | 5'-GATCTGCTGTGCCGTGATCT-3' |
| JPT2-Forward (mouse) | 5'-CAAGCTGGCAAGTCGGGTT-3' |
| JPT2-Reverse (mouse) | 5'-GCACAGGAGTCGATTCATCAAAG-3' |
| PSMC6-Forward (mouse) | 5'-ATGGCGGACCCTAGAGATAAG-3' |
| PSMC6-Reverse (mouse) | 5'-TCACCTACAATCTGTCCAACACT-3' |
| HUWE1-Forward (mouse) | 5'-TGCTTGTATGTGATAGGCCAGA-3' |
| HUWE1-Reverse (mouse) | 5'-AGCGGAGTCCTCTTGTCAGAT-3' |
| MAP4K4-Forward (mouse) | 5'-CTGGCCGCCATCAAGGTTAT-3' |
| MAP4K4-Reverse (mouse) | 5'-AGCACCATAGTACGTGGCAAT-3' |
| SPAG9-Forward (mouse) | 5'-AGGTTGCCCAAGAGACTAGGA-3' |
| SPAG9-Reverse (mouse) | 5'-AGGAGTGGATTCAATGATTGCTT-3' |
| RPL37-Forward (mouse) | 5'-CTCGGAGGTTACGGGACTC-3' |
| RPL37-Reverse (mouse) | 5'-CTTGCCCTCGTAGGTAATGGG-3' |
| CAND1-Forward (mouse) | 5'-CTTGGTCACATTCAATTCTGCG-3' |
| CAND1-Reverse (mouse) | 5'-GACCGTCATCAACCGTGTGTT-3' |
| FAM228A-Forward (mouse) | 5'-AAGTGTCTTCATCGTGTCTGTTT-3' |
| FAM228A-Reverse (mouse) | 5'-TCATGTGTTTTTCCTCTGCCAT-3' |
| HSPE1-Forward(mouse) | 5'-AGTTTCTTCCGCTCTTTGACAG-3' |
| HSPE1-Reverse (mouse) | 5'-TGCCACCTTTGGTTACAGTTTC-3' |
| FAM193A-Forward mouse) | 5'-CTCAGGCACACCACTTTGTCT-3' |
| FAM193A-Reverse (mouse) | 5'-TATGCAGTTCCCATGTCACCT-3' |
| USP10-Forward (mouse) | 5'-AACCCACAGTATATCTTTGGCG-3' |
| USP10-Reverse (mouse) | 5'-CCCTCACTAGGTTCGATGACTTC-3' |
| RPL31-Forward (mouse) | 5'-CGTTCTGCCATCAACGAGGT-3' |
| RPL31-Reverse (mouse) | 5'-TCCGAATTTCTTTGAGTGCCC-3' |
