# Supplementary material for: Sperm-Associated Antigen 9 Promotes Influenza A Virus-Induced Cell Death via the c-Jun N-Terminal Kinase Signaling Pathway
Source: mBio. 2022 May 31;13(3):e00615-22. doi: 10.1128/mbio.00615-22 (PMC9239253; doi:10.1128/mbio.00615-22)
Supplement: TABLE S2 [file mbio.00615-22-s0002.docx]

**Table S2.** The sequence of siRNA was used for gene knockdown

| siRNA targeting gene name | sequence |
| --- | --- |
| Negative control siRNA | 5´- AAUUCUCCGAACGUGUCACGU -3´ |
| JNK1(human) | 5´- AAGCCCAGUAAUAUAGUAGUA -3´ |
| JNK2(human) | 5´- AAGCCGTCCTTTTCAGAACCA -3´ |
| JNK1(mouse) | 5´GGAGUUAGAUCAUGAAAGAAU-3´ |
| JNK2(mouse) | 5´AGAGCUAAUUUACAAAGAAGU-3´ |
| UPF1(mouse) | 5´-GCAAGAAGUGGUUCUGCAAUG-3´ |
| JPT2(mouse) | 5´-GAAGAAGGUAUUUCUUCAAGC-3´ |
| PSMC6(mouse) | 5´-GAUUGUAGGUGAAGUGCUAAA-3´ |
| HUWE1(mouse) | 5´-CGAGAGAUGUUCAACCCUAUG-3´ |
| MAP4K4(mouse) | 5´-CGUUCAUGAUGAUGUAGAAAG-3´ |
| SPAG9(mouse) | 5´-AGAUGUGUACAAAGAUCAAAU-3´ |
| RPL37(mouse) | 5´-GCUAAGAGACGAAACACUACC-3´ |
| CAND1(mouse) | 5´-GGUUCCCAACAUUGUUAAAGC-3´ |
| FAM228A(mouse) | 5´-GAGAUUGGAUACAUAUUUACA-3´ |
| HSPE1(mouse) | 5´-GCACCAAAGUAGUUCUAGAUG-3´ |
| FAM193A(mouse) | 5´-CCACAGAGGUACAGCUCAAAC-3´ |
| USP10(mouse) | 5´-CGAGUUUGGUGUAGAUGAAGU-3´ |
| RPL31(mouse) | 5´-CGAGAAUACACCAUCAACAUU-3´ |
